# Discovery of Matrinic Thiadiazole Derivatives as a Novel Family of Anti-Liver Fibrosis Agents via Repression of the TGFβ/Smad Pathway

**DOI:** 10.3390/molecules23071644

**Published:** 2018-07-05

**Authors:** Tianyu Niu, Weixiao Niu, Yunyang Bao, Ting Liu, Danqing Song, Yinghong Li, Hongwei He

**Affiliations:** Institute of Medicinal Biotechnology, Chinese Academy of Medical Sciences and Peking Union Medical College, Beijing 100050, China; niutianyubasn@163.com (T.N.); niuweixiao@126.com (W.N.); 17714333891@163.com (Y.B.); lutyliu@126.com (T.L.); songdanqingsdq@hotmail.com (D.S.)

**Keywords:** liver fibrosis, matrinic, thiadiazole, structure-activity relationship, COL1A1, TGFβ/Smad pathway

## Abstract

A series of novel matrinic thiadiazole derivatives were designed, synthesized and evaluated for their inhibitory effect on COL1A1 promotor. The SAR indicated that: (i) the introduction of a thiadiazole on the 11-side chain was beneficial for activity; (ii) a 12-*N*-benzyl moiety was favorable for activity. Among them, compound **6n** displayed a high activity with an inhibitory rate of 39.7% at a concentration of 40 μM. It also effectively inhibited the expression of two representative collagen proteins (COL1A1 and α-SMA) on both the mRNA and protein levels and showed a high safety profile in vivo, indicating its great promise as an anti-liver fibrosis agent. Further study indicated that it might repress hepatic fibrogenesis via the TGFβ/Smad pathway. This study provided powerful information for further strategic optimization and the top compound **6n** was selected for further study as an ideal liver fibrosis lead for next investigation.

## 1. Introduction

Liver fibrosis is a general stage in the process of cirrhosis, which is characterized by the excessive accumulation of extracellular matrix (ECM) and abnormal hyperplasia of intrahepatic connective tissue [1]. Fibrillation is the scarring response to chronic liver injury [2]. It can be controlled in the early phase after effective treatment, but most often, fibrous septa, pseudolobules or nodules would form and eventually develop into liver cirrhosis. Unfortunately, to date, treatments for liver fibrosis are quite limited and therefore, there is still an urgent need to develop new effective anti-liver fibrosis candidates.

The activation and proliferation of hepatic stellate cells (HSCs) leads to the excess production and abnormal deposition of ECM components [3,4], as indicated in the cytological basis for liver fibrosis [5].Transforming growth factor β1 (TGFβ1) is closely related to liver fibrosis, and it is one of the most important fibrotic cytokines known so far [6]. It promotes the production of liver fibrosis mainly by activating HSCs and up-regulating the secretion of collagen, especially collagen type I (COL1) and α-smooth muscle actin (α-SMA). Since the transcription of key gene collagen type I α1 chain (COL1A1) for liver fibrosis could be activated by TGFβ1 through TGFβ/Smad pathway [7], a high-throughput drug screening cell model based on COL1A1 promotor was established earlier in our group, in which the activity of COL1A1 promotor and luciferase reporter gene could be elevated by TGFβ1, and inhibited by candidate agents [8].

**Figure 1:**
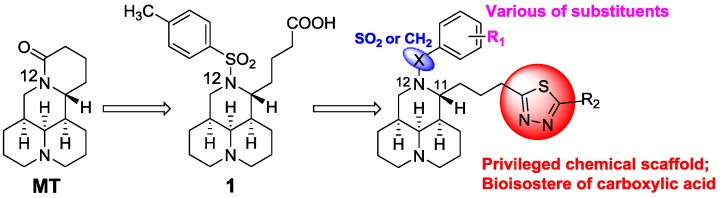
Chemical structures of matrine and the lead compound **1**, and structure modification strategy.

Our group has been working on the discovery of innovative candidates agents with novel structure skeletons from Chinese natural products such as matrine (MT) [9,10,11]. Since MT is an anti-liver fibrosis monomer [12], the matrinic acid compound library constructed in our lab [9,10,13] was screened by the high-throughput screening model based on COL1A1 promotor as mentioned above, taking epigallocatechin gallate (EGCG) as the positive control [14]. To our great delight, the hit compound 12-*N*-*p*-methyl benzenesulfonyl matrinic acid (**1**, Figure 1) [15] had been identified to display a reasonable inhibitory effect against COL1A1 at a rate of 18.5% at the concentration of 40 μM. It was also worth mentioning that compound **1** possesses a special tricyclic flexible scaffold, and appealing druglike characteristics. These studies prompted our interest in continuing our structure-activity relationship (SAR) study of this compound class taking compound **1** as the lead, in an effort to develop and discover novel anti-liver fibrosis candidates.

The 1,3,4-thiadiazole group is recognized as a bioisostere of the carboxyl group [16], and also a privileged chemical scaffold [17,18,19], therefore, in the present study, the carboxyl was replaced with 1,3,4-thiadiazole group on the 11-side chain, whereby a series of novel tricyclic matrinic thiadiazole derivatives was generated and evaluated for their anti-COL1A1 activity. Herein, we describe the synthesis of 19 novel thiadiazole derivatives, SAR analysis, as well as in vivo safety profile and primary mechanism of action of key compounds.

## 2. Results and Discussion

### 2.1. Chemistry

**Scheme 1:**
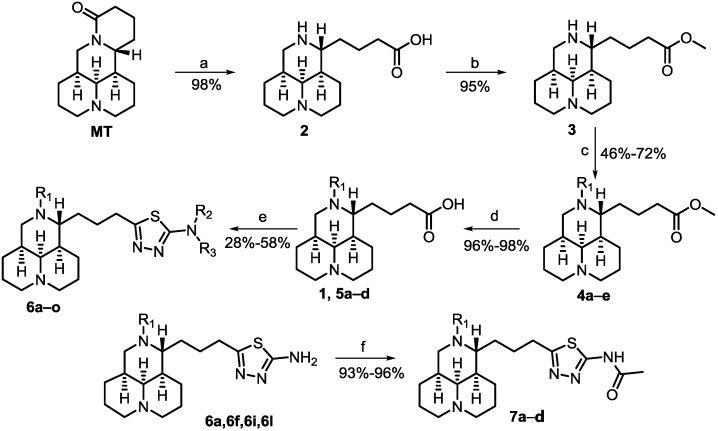
*Reagents and conditions*: (a) 5 N NaOH, reflux, 9 h; 10 N HCl, pH = 3–5; (b) 2 N MeOH/HCl, reflux, 2 h; (c) Substituted benzensulfonyl chloride or benzyl bromide, TEA or K_2_CO_3_, CH_2_Cl_2_ or MeCN, r.t., 6–8 h; (d) 5 N NaOH, reflux, 6 h; 10 N HCl, pH = 7–7.5; (e) P_2_O_5_/MsOH (1:5), thiosemicarbazide, 70 °C, 8 h; (f) Acetyl chloride, TEA, THF, r.t., 0.5 h.

The semi-synthetic routes of all target compounds were outlined in Scheme 1, taking MT as the starting material. 12-*N*-substituted matrinic acids **1**, **5a**–**d** were obtained in high yields of 42–66% by a four-step procedure including hydrolysis, esterification, 12-*N* substitution and hydrolysis instead of the original diphenyldiazomethane protection method [9,15]. Then a series of matrinic thiadiazole derivatives **6a**–**o** were obtained by the cyclization of **1** or **5a**–**d** with thiosemicarbazide or various of *N*-substituted thiosemicarbazides in the presence of phosphorus pentoxide/methanesulfonic acid (1:5) in yields of 28–58% [20,21]. The acylation of **6a**, **6f**, **6i** and **6l** in triethylamine generated compounds **7a**–**d** with yields of 93–96%. The desired products were purified with flash column chromatography on silica gel using dichloromethane/methanol or petroleum/ethyl acetate as eluents.

### 2.2. Inhibition of COL1A1 Promotor in Human Hepatic Stellate LX-2 Cells by Target Compounds

A single luciferase reporter gene detection model was applied to screen the inhibitory effects towards COL1A1 promotor of all target compounds in human hepatic stellate LX-2 cells at the concentration of 40 μM, taking EGCG and compound **1** as the positive controls. The LX2-COL monoclone cell line was treated simultaneously with TGFβ1 and a target compound [13]. The cytotoxicity was determined using MTT assay in HepG2 cells. The structures and inhibitory effects (%) of all target compounds are shown in Table 1.

Taking **1** as the lead, the carboxyl group was retained, and the SAR investigation was initiated with the variation of 12-*N* substitution, by which 4 matrinic acid derivatives **5a**–**d** were obtained. As depicted in Table 1, the replacement of the methyl group on the benzene ring with a trifluoromethyl group (compound **5a**) or the replacement of a benznesulfonyl with a benzyl motif (compounds **5b**–**d**) caused a significant decrease in activity.

The carboxyl group at the end of the 11-side chain was then replaced by its 1,3,4-thiadiazole bioisostere, and a series of target matrinic thiadiazole compounds was prepared and evaluated. To start, the 12-*N*-*p*-methylbenzenesulfonyl was retained, and five matrinic aminothiadiazoles **6a**–**e** were obtained. Compound **6a** showed higher inhibitory effects than **6b**–**d** and the lead **1**, which indicated that 5-amino-1,3,4-thiadiazole motif was a beneficial group, and the introduction of an extra alkyl group on the amino group was not helpful for activity in this series. In the meantime, the introduction of a phenyl group into the amino (compound **6e**) resulted in an obvious increase in activity. It was indicated that the aminothiadiazole motif was more beneficial, which might result from the smaller spatial hindrance. Then, the 12-*N*-substituent was replaced with a *p*-trifluoromethyl benzenesulfonyl moiety to prepare target compounds **6f**–**h**, which displayed decreased activities in varied degrees, as anticipated.

Next, the aminothiadiazole motif was retained and the 12-***N***-benzenesulfonyl was replaced with a 12-***N***-benzyl group to generate the corresponding compounds **6i**–**o**. To our great delight, most of them showed a significant improvement in activity, and compounds **6i**, **6l** and **6n** gave inspiring inhibitory rates of 47.8%, 38.7% and 39.7%, respectively, significantly higher than that of **1** (18.5%) and EGCG (27.5%). These results suggested that benzyl was a favorable 12-*N* substitution.

Finally, an acyl group was condensed to the amino group of **6a**, **6f**, **6i** and **6l** to generate **7a**–**d**, respectively, and 12-***N***-benzenesulfonyl compounds **7a** and **7b** displayed higher activity than their amino counterparts **6a** and **6f**, while the 12-***N***-benzyl compounds **7c** and **7d** displayed lower activity than their counterparts **6i** and **6l**.

Five target compounds **6a**, **6e**, **6i**, **6l** and **6n** with the highest activity were selected as the representative compounds to further evaluate their cytotoxicities. As indicated in Table 1, compounds **6a**, **6i**, **6l** and **6n** displayed high cellular safety with the concentration of half cellular toxicity (CC_50_) value of over 320 μM, comparable to that of **1**, while compound **6e** displayed a high cellular toxicity with a CC_50_ value of over 82.6 μM, indicating that the introduction of a benzylamino to the thiadiazole motif might contribute to a high cytotoxicity, which might result from its high lipophilicity.

### 2.3. Inhibition Effects of COL1A1 on RNA and Protein Levels by Key Compounds

**Figure 2:**
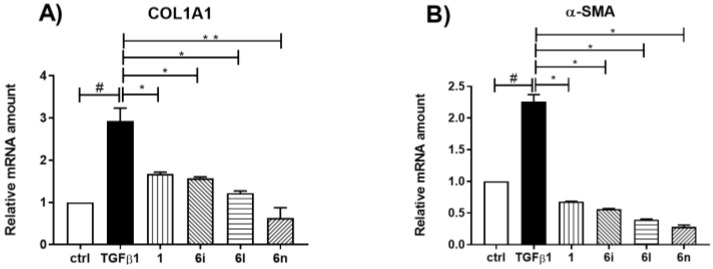
Effects of compounds **6i**, **6l** and **6n** inhibiting (**A**) COL1A1, (**B**) α-SMA mRNA levels in LX-2 cells. Data were analyzed by Real-time PCR and presented as the mean ± SEM, (#) *p* < 0.05 as compared to that of control group; (*) *p* < 0.05, (**) *p* < 0.01 as compared to that of TGF-β1 group. The mRNA expression levels were normalized against GAPDH.

**Figure 3:**
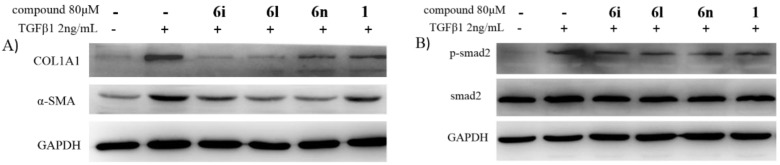
(**A**) Effects of compounds **6i**, **6l** and **6n** on inhibiting fibrogenetic COL1A1 and α-SMA protein levels in LX-2 cells; (**B**) Effects of compounds **6i**, **6l** and **6n** on p-smad2 and smad2 levels in LX-2 cells. Data were analyzed by western blot assay. The protein expression levels were normalized against GAPDH.

Next, compounds **6i**, **6l** and **6n** were chosen as the key compounds to verify their inhibitory effects against COL1A1. LX-2 cells were stimulated with TGFβ1 (2 ng/mL), and then treated with compounds **6i**, **6l** and **6n** (80 μM) respectively, taking **1** as the control. Their anti-COL1A1 effects were evaluated on RNA level by real-time PCR amplification. As indicated in Figure 2A, all three compounds **6i**, **6l** and **6n** could significantly reduce COL1A1 mRNA level with the inhibitory rates of 46.1%,58.2% and 78.4%, respectively, prevailing over that of **1** (41.9%) to varying degrees. Then their anti-COL1A1 effects were investigated on the protein level by western blot assays. As indicated in Figure 3A, **6i**, **6l** and **6n** could significantly reduce the COL1A1 protein level with inhibition rates of 65.0%, 99.3% and 47.6%, respectively, significantly higher than that of **1** (16.2%). These results suggested that these matrinic thiadiazole compounds could effectively reduce COL1A1 on both the mRNA and protein levels.

### 2.4. Inhibition Effects of α-SMA on RNA and Protein Levels by Key Compounds

α-SMA is a more commonly recognized indicator for liver fibrosis, and it could be induced by a stimulation of TGFβ1 [22], therefore, the amounts of α-SMA were also evaluated. In the same experiments as illustrated above, **6i**, **6l** and **6n** significantly reduced α-SMA mRNA levels with inhibitory rates of 75.1%, 82.5% and 87.6% respectively, superior to that of **1** (69.7%), as indicated in Figure 2B. Compounds **6i**, **6l** and **6n** significantly reduced the α-SMA protein level with inhibition rates of 64.9%, 93.8% and 104.1%, respectively, significantly superior to that of 1 (28.9%), as indicated in Figure 3A. These results disclosed that these kind of compounds also inhibited fibrogenetic α-SMA on both mRNA and protein levels.

### 2.5. Action on TGFβ/Smad Pathway of Key Compounds

**Figure 4:**
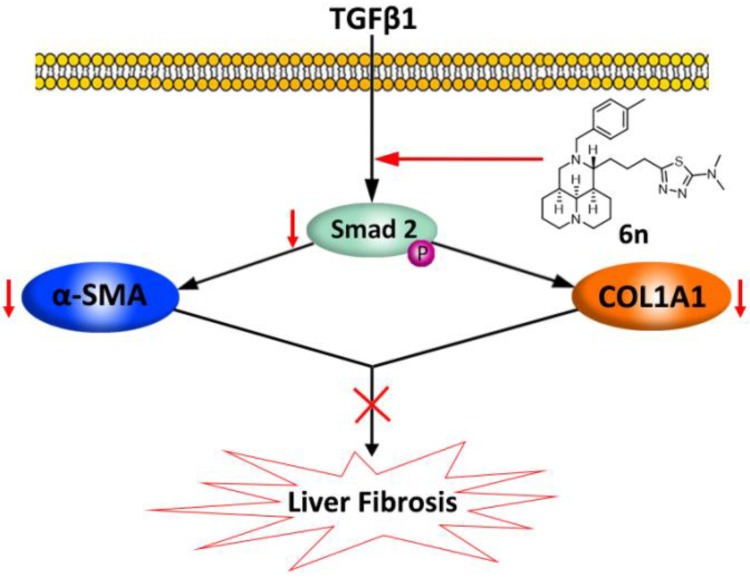
Compound **6n** exerted the anti-liver fibrotic activity via repressing the Smad/TGFβ pathway.

Considering the key role of TGFβ/Smad pathway in activating COL1A1, the effects of the three compounds on Smad and phosphorylated Smad were then evaluated. As indicated in Figure 2B, **6i**, **6l** and **6n** did not affect the Smad level, while they repressed the phosphorylation level of Smad2 significantly, therefore, it was speculated that they might exert anti-liver fibrotic activity via repressing the TGFβ/Smad pathway, as depicted in Figure 4. Among them, compound **6n** showed the highest inhibition rate of 49.8% as anticipated, and it was selected for the next investigation.

### 2.6. Safety Profile of Key Compounds

To evaluate the safety profile of **6n**, an acute toxicity test was performed in Kunming mice. Compound **6n** was given orally in a single-dosing experiment at 125, 250 or 500 mg·kg^−1^, respectively. The mice were closely monitored for 7 days. To our delight, it gave the median lethal dose (LD_50_) value over 500 mg·kg^−1^, indicating a good safety profile in vivo.

## 3. Experimental Section

### 3.1. Apparatus, Materials, and Analysis Reagents

All chemical reagents and anhydrous solvents were obtained from commercial sources and used without further purification. Melting points (mp) were obtained with a MP90 melting point apparatus and was uncorrected (Mettler-Toledo, Greifensee, Switzerland). Specific rotations were obtained with Rudolph Autopol IV automatic polarimeter (Jasco Products Company, Oklahoma City, OK, USA). ^1^H- and ^13^C-NMR spectra were recorded on Avance (600 MHz and 500 MHz for ^1^H-NMR; 151 MHz and 126 MHz for ^13^C-NMR) spectrometers (Bruker, Zürich, Switzerland). The ^1^H chemical shifts were referenced to the solvent peak: DMSO-*d*_6_ (2.49 ppm) and CDCl_3_ (7.26 ppm) and the ^13^C chemical shifts were referenced to the solvent peak: DMSO-*d*_6_ (40.5 ppm) and CDCl_3_ (77.4 ppm). ESI high-resolution mass spectra (HRMS) were recorded on an AutospecUltima-TOF spectrometer (Micromass UK Ltd., Manchester, UK). The method of the HPLC analysis was as follows: column, Inertsustain C18, 250 mm × 4.6 mm, id; flow rate, 1.0 mL/min; mobile phase, acetonitrile/water (0.01 mol·L^−1^ KH_2_PO_4_ in water, pH = 4.0) = 5:95; Flash column chromatography was performed on Combiflash Rf 200 (Teledyne, Lincoln, NE, USA), particle size 0.038 mm. The target compounds **1** and **5a**–**d** were reported previously [9,15,23], but **1** and **5b**–**d** were obtained in this manuscript by a new method with improved yields.

### 3.2. Chemistry

#### 3.2.1. General Procedure for the Synthesis of Compounds **1**, **5b**–**d**

Matrine (5.0 g, 20 mmol) was added to 5 N NaOH solution (30 mL), and the reaction mixture was refluxed for 9 h, cooled to room temperature and then acidified with HCl (2 N) to pH 2–3. The solvent was removed in vacuo and the residue was dissolved with 2% HCl in methanol and then refluxed for 2 h. The solvent was removed under reduced pressure to give a crude product, which was purified by recrystallization from ethanol to achieve intermediate **3** (6.6 g, 93.1%). To a suspension of compound **3** (5.0 g, 14 mmol) in dichloromethane (50 mL), triethylamine (4.34 g, 43 mmol) and substituted benzenesulfonyl chloride (17 mmol) or substituted benzyl bromide (17 mmol) were added, the reaction mixture was stirred at room temperature for 6 h until TLC analysis showed completion of the reaction. The reaction mixture was then washed by water (50 mL), saturated aqueous ammonium chloride (50 mL) and saturated aqueous sodium chloride (50 mL) subsequently, dried over anhydrous sodium sulfate and concentrated under reduced pressure. The residue was purified by recrystallization from methanol to give **4**. Compound **4** (10 mmol) were refluxed in 5 N NaOH solution (30 mL) for 4 h, cooled and acidified with HCl (2 N) to pH 4–5. The solvent was removed by condensation, and the residue was suspended in methanol, and the precipate was filtered off. The filtrate was evapotared to give a crude. The desired compounds (**1** or **5b**–**d**) were gained by flash column chromatography purification on silica gel with dichloromethane/methanol as the eluent.

*12-N-p-Methylbenzenesulfonyl matrinic acid* (**1**). Total yield: 64%, m.p.: 239–240 °C (Total yiled: 6%, m.p.: 239–241 °C [15]).

*12-N-Benzyl matrinic acid* (**5b**). Total yield: 41%, m.p.: 95–96 °C (Total yield: 15%, m.p.: 95–96 °C [15]).

*12-N-p-Methylbenzyl matrinic acid* (**5c**). Total yield: 46%, m.p.: 106–108 °C (Total yield: 34%, m.p.: 106–108 °C [9]).

*12-N-p-Bromobenzyl matrinic acid* (**5d**). Total yield: 59%, m.p.: 134–135 °C (Total yield: 27%, m.p.: 133–135 °C [9]).

#### 3.2.2. General Procedure for the Synthesis of Compounds **6a**–**o**


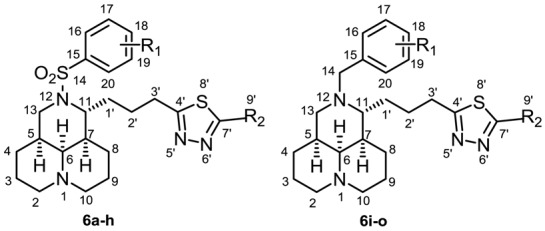


A mixture of **1** or **5a**–**d** (2.5 mmol), thiosemicarbazide or *N*-substituted thiosemicarbazide (2.5 mmol), methanesulfonic acid (12.5 mmol) and phosphorus pentoxide (2.5 mmol) were heated at 70 °C for 8 h. The reaction mixture was poured into ice water (40 mL) and neutralized with aqueous ammonia to pH 8. The solution was extracted with dichloromethane (50 mL), and the organic layer was washed by saturated aqueous sodium chloride (50 mL), dried over anhydrous sodium sulfate, and concentrated under reduced pressure. The residue was purified by flash column chromatography on silica gel with dichloromethane/methanol or petroleum ether/ethyl acetate as the eluent.

*12-N-p-Methlybenzenesulfonyl-3′-(5-amino-1,3,4-thiadiazol-2-yl)-matrinic propane* (**6a**). Compound **1** (1.04 g, 2.5 mmol) was treated with thiosemicarbazide (0.23 g, 2.5 mmol) according to the general procedure, then purified by flash column chromatography with dichloromethane/methanol as the eluent to give the desired product **6a** as a white solid, yield: 40.9%; m.p.: 237 °C (dec.); ${\left[\alpha \right]}_{589}^{25}$ = −6.16; ^1^H-NMR (500 MHz, DMSO-*d*_6_) δ 7.68 (d, *J* = 8.2 Hz, 2H, 16-CH, 20-CH), 7.38 (d, *J* = 8.2 Hz, 2H, 17-CH, 19-CH), 7.00 (s, 2H, 9′-NH_2_), 3.42–3.36 (m, 2H, 3′-CH_2_), 3.16–3.08 (m, 1H, 6-CH), 2.73–2.69 (m, 1H, 5-CH), 2.65–2.61 (m, 1H, 7-CH), 2.59–2.56 (m, 1H, 11-CH), 2.38 (s, 3H, Ph-CH_3_), 1.95–1.90 (m, 1H, 1′a-CH), 1.87–1.80 (m, 2H, 2′-CH_2_), 1.76–1.61 (m, 6H, 2-CH_2_, 10-CH_2_, 13-CH_2_), 1.51–1.45 (m, 2H, 4-CH_2_), 1.39–1.23 (m, 7H, 3-CH_2_, 8-CH_2_, 9-CH_2_, 1′b-CH); ^13^C-NMR (126 MHz, DMSO-*d*_6_) δ 168.2 (7′-C), 158.2 (4′-C), 142.9 (18-C), 136.7 (15-C), 129.5 (17-CH, 19-CH), 127.2 (16-CH, 20-CH), 62.3 (6-CH), 56.6 (11-CH), 56.2 (2-CH_2_), 56.1 (10-CH_2_), 47.1 (13-CH_2_), 38.6 (7-CH), 34.0 (5-CH), 29.7 (1′-CH_2_), 29.6 (3′-CH_2_), 27.6 (4-CH_2_), 27.5 (8-CH_2_), 24.8 (2′-CH_2_), 21.1 (Ph-CH_3_), 20.3 (3-CH_2_), 20.2 (9-CH_2_); ESI-HRMS: *m*/*z* Calcd for C_23_H_34_O_2_N_5_S_2_ [M + H]^+^, 476.2148; Found, 476.2149; HPLC: *t_R_* = 10.29 min, normalization method purity 97.11%.

*12-N-p-Mmethlybenzenesulfonyl-3′-(N-methyl-5-amino-1,3,4-thiadiazol-2-yl)-matrinic propane* (**6b**). Compound **1** (1.04 g, 2.5 mmol) was treated with 4-methyl-3-thiosemicarbazide (0.26 g, 2.5 mmol) according to the general procedure, then purified by flash column chromatography with dichloromethane/methanol as the eluents to give to give the desired product **6b** as a white solid, yield: 33.4%; m.p.: 169–170 °C; ${\left[\alpha \right]}_{589}^{25}$ = −7.98; ^1^H-NMR (500 MHz, DMSO-*d*_6_) δ 7.68 (d, *J* = 7.9 Hz, 2H, 16-CH, 20-CH), 7.49 (q, *J* = 4.8 Hz, 1H, 9′-NH), 7.38 (d, *J* = 7.9 Hz, 2H, 17-CH, 19-CH), 3.43–3.35 (m, 2H, 3′-CH_2_), 3.14–3.09 (m, 1H, 6-CH), 2.83 (d, *J* = 4.8 Hz, 3H, 9′-NCH_3_), 2.76–2.70 (m, 1H, 5-CH), 2.67–2.61 (m, 1H, 7-CH), 2.57 (m 1H, 11-CH), 2.38 (s, 3H, Ph-CH_3_), 1.95–1.90 (m, 1H, 1′a-CH), 1.86–1.80 (m, 2H, 2′-CH_2_), 1.75–1.62 (m, 6H, 2-CH_2_, 10-CH_2_, 13-CH_2_), 1.49–1.23 (m, 9H, 3-CH_2_, 4-CH_2_, 8-CH_2_, 9-CH_2_, 1′b-CH ); ^13^C-NMR (126 MHz, DMSO-*d*_6_) δ 169.1 (7′-C), 157.6 (4′-C), 142.9 (18-C), 136.7 (15-C), 129.5 (17-CH, 19-CH), 127.2 (16-CH, 20-CH), 62.3 (6-CH), 56.6 (11-CH), 56.1 (2-CH_2_), 56.0 (10-CH_2_), 47.0 (13-CH_2_), 38.6 (7-CH), 34.0 (5-CH), 31.2 (9′-NCH_3_), 29.7 (1′-CH_2_), 29.6 (3′-CH_2_), 27.5 (4-CH_2_), 27.4 (8-CH_2_), 24.9 (2′-CH_2_), 21.0 (Ph-CH_3_), 20.3 (3-CH_2_), 20.2 (9-CH_2_); ESI-HRMS: *m*/*z* Calcd for C_24_H_36_O_2_N_5_S_2_ [M + H]^+^, 490.2304; Found, 490.2304; HPLC: *t_R_* = 10.78 min, normalization method purity 98.74%.

*12-N-p-Methylbenzenesulfonyl-3′-(N-iso-propyl-5-amino-1,3,4-thiadiazol-2-yl)-matrinic propane* (**6c**). Compound **1** (1.04 g, 2.5 mmol) was treated with 4-iso-propyl-3-thiosemicarbazide (0.33 g, 2.5 mmol) according to the general procedure, then purified by flash column chromatography with dichloromethane/methanol as the eluents to give the desired product **6d** as a white solid, yield: 34.1%; m.p.: 142–143 °C; ${\left[\alpha \right]}_{589}^{25}$ = −15.79; ^1^H-NMR (600 MHz, DMSO-*d*_6_) δ 7.68 (d, *J* = 8.1 Hz, 2H, 16-CH, 20-CH), 7.44 (d, *J* = 7.2 Hz, 1H, 9′-NH), 7.38 (d, *J* = 8.1 Hz, 2H, 17-CH, 19-CH), 3.77–3.69 (m, 1H, 9′-NCH), 3.42–3.38 (m, 2H, 3′-CH_2_), 3.13 (t, *J* = 11.6 Hz, 1H, 6-CH), 2.74–2.70 (m, 1H, 5-CH), 2.66–2.61 (m, 1H, 7-CH), 2.58–2.53 (m, 1H, 11-CH), 2.38 (s, 3H, Ph-CH_3_), 1.94 (m, 1H, 1′a-CH), 1.86–1.80 (m, 2H, 2′-CH_2_), 1.75–1.61 (m, 6H, 2-CH_2_, 10-CH_2_, 13-CH_2_), 1.49–1.46 (m, 2H, 4-CH_2_), 1.39–1.23 (m, 7H, 3-CH_2_, 8-CH_2_, 9-CH_2_, 1′b-CH), 1.17 (d, *J* = 6.4 Hz, 6H, NC(CH_3_)_2_); ^13^C-NMR (151 MHz, DMSO-*d*_6_) δ 167.4 (7′-C), 157.2 (4′-C), 142.9 (18-C), 136.7 (15-C), 129.5 (17-CH, 19-CH), 127.2 (16-CH, 20-CH), 62.3 (6-CH), 56.6 (11-CH), 56.1 (2-CH_2_), 56.0 (10-CH_2_), 47.0 (13-CH_2_), 46.5 (NCH), 38.6 (7-CH), 34.0 (5-CH), 29.8 (1′-CH_2_), 29.5 (3′-CH_2_), 27.5 (4-CH_2_), 27.4 (8-CH_2_), 24.9 (2′-CH_2_), 22.2 (NC(CH_3_)_2_), 21.0 (Ph-CH_3_), 20.3 (3-CH_2_), 20.2 (9-CH_2_); ESI-HRMS: *m*/*z* Calcd for C_26_H_40_O_2_N_5_S_2_ [M + H]^+^, 518.2617; Found, 518.2607; HPLC: *t_R_* = 12.11 min, normalization method purity 97.99%.

*12-N-p-Methylbenzenesulfonyl-3′-(N,N-dimethyl-5-amino-1,3,4-thiadiazol-2-yl)-matrinic propane* (**6d**). Compound **1** (1.04 g, 2.5 mmol) was treated with 4,4-dimethyl-3-thiosemicarbazide (0.31 g, 2.5 mmol) according to the general procedure, then purified by flash column chromatography with dichloromethane/methanol as the eluents to give the desired product **6e** as a white solid, yield: 24.2%; m.p.: 171–172 °C; ${\left[\alpha \right]}_{589}^{25}$ = −9.66; ^1^H-NMR (500 MHz, DMSO-*d*_6_) δ 7.68 (d, *J* = 8.2 Hz, 2H, 16-CH, 20-CH), 7.39 (d, *J* = 8.2 Hz, 2H, 17-CH, 19-CH), 3.45–3.36 (m, 2H, 3′-CH_2_), 3.18–3.07 (m, 1H, 6-CH), 3.03 (s, 6H, N(CH_3_)_2_), 2.81–2.72 (m, 1H, 5-CH), 2.72–2.63 (m, 1H, 7-CH), 2.57 (d, *J* = 11.4 Hz, 1H, 11-CH), 2.38 (s, 3H, Ph-CH_3_), 1.94 (m, 1H, 1′a-CH), 1.90–1.79 (m, 2H, 2′-CH_2_), 1.78–1.60 (m, 6H, 2-CH_2_, 10-CH_2_, 13-CH_2_), 1.54–1.18 (m, 9H, 3-CH_2_, 4-CH_2_, 8-CH_2_, 9-CH_2_, 1′b-CH); ^13^C-NMR (151 MHz, DMSO-*d*_6_) δ 171.2 (7′-C), 158.5 (4′-C), 142.8 (18-C), 136.8 (15-C), 129.5 (17-CH, 19-CH), 127.1 (16-CH, 20-CH), 62.3 (6-CH), 56.6 (11-CH), 56.1 (2-CH_2_), 56.0 (10-CH_2_), 47.0 (13-CH_2_), 41.1 (N(CH_3_)_2_), 38.7 (7-CH), 34.0 (5-CH), 29.7 (1′-CH_2_), 29.6 (3′-CH_2_), 27.5 (4-CH_2_), 27.4 (8-CH_2_), 25.1 (2′-CH_2_), 21.0 (Ph-CH_3_), 20.3 (3-CH_2_), 20.2 (9-CH_2_); ESI-HRMS: *m*/*z* Calcd for C_25_H_38_O_2_N_5_S_2_ [M + H]^+^, 504.2441; Found, 504.2447; HPLC: *t_R_* = 11.59 min, normalization method purity 98.45%.

*12-N-p-Methylbenzenesulfonyl-3′-(N-phenyl-5-amino-1,3,4-thiadiazol-2-yl)-matrinic propane* (**6e**). Compound **1** (1.04 g, 2.5 mmol) was treated with 4-phenyl-3-thiosemicarbazide (0.42 g, 2.5 mmol) according to the general procedure, then purified by flash column chromatography with petroleum ether/ethyl acetate as the eluents to give the desired product **6c** as a white solid, yield: 48.2%; m.p.: 193–194 °C; ${\left[\alpha \right]}_{589}^{25}$ = −13.89; ^1^H-NMR (500 MHz, DMSO-*d*_6_) δ 10.26 (s, 1H, 9′-NH), 7.68 (d, *J* = 8.0 Hz, 2H, 16-CH, 20-CH), 7.60 (d, *J* = 8.0 Hz, 2H, 17-CH, 19-CH), 7.38 (d, *J* = 7.5 Hz, 2H, 2 × 9′-NCH_arom_), 7.33 (t, *J* = 7.5 Hz, 2H, 2 × 9′-NCH_arom_), 6.97 (t, *J* = 7.5 Hz, 1H, 9′-NCH_arom_ ), 3.43–3.38 (m, 2H, 3′-CH_2_), 3.13 (t, *J* = 11.6 Hz, 1H, 6-CH), 2.87–2.81 (m, 1H, 5-CH), 2.79–2.72 (m, 1H, 7-CH), 2.57 (d, *J* = 11.1 Hz, 1H, 11-CH), 2.35 (s, 3H, Ph-CH_3_), 1.93 (m, 1H, 1′a-CH), 1.86–1.83 (m, 2H, 2′-CH_2_), 1.75–1.68 (m, 6H, 2-CH_2_, 10-CH_2_, 13-CH_2_), 1.56–1.23 (m, 9H, 3-CH_2_, 4-CH_2_, 8-CH_2_, 9-CH_2_, 1′b-CH); ^13^C-NMR (151 MHz, DMSO-*d*_6_) δ 163.9 (7′-C), 159.5 (4′-C), 142.8 (18-C), 140.8 (9′N-Ph), 136.8 (15-C), 129.5 (17-CH, 19-CH), 129.0 (9′N-Ph), 127.1 (16-CH, 20-CH), 121.6 (9′N-Ph), 117.1 (9′N-Ph), 62.3 (6-CH), 56.6 (11-CH), 56.1 (2-CH_2_), 56.0 (10-CH_2_), 46.9 (13-CH_2_), 38.8 (7-CH), 34.0 (5-CH), 29.8 (1′-CH_2_), 29.4 (3′-CH_2_), 27.6 (4-CH_2_), 27.5 (8-CH_2_), 24.9 (2′-CH_2_), 21.0 (Ph-CH_3_), 20.3 (3-CH_2_), 20.2 (9-CH_2_); ESI-HRMS: *m*/*z* Calcd for C_29_H_38_O_2_N_5_S_2_ [M + H]^+^, 552.2461; Found, 552.2462; HPLC: *t_R_* = 13.39 min, normalization method purity 96.69%.

*12-N-p-Ttrifluoromethlybenzenesulfonyl-3′-(5-amino-1,3,4-thiadiazol-2-yl)-matrinic propane* (**6f**). Compound **5a** (1.19 g, 2.5 mmol) was treated with thiosemicarbazide (0.23 g, 2.5 mmol) according to the general procedure, then purified by flash column chromatography with dichloromethane/methanol as the eluents to give the desired product **6f** as a white solid, yield: 48.0%; m.p.: 206 °C (dec.); ${\left[\alpha \right]}_{589}^{25}$ = −0.65; ^1^H-NMR (500 MHz, CDCl_3_) δ 7.96 (d, *J* = 8.2 Hz, 2H, 16-CH, 20-CH), 7.73 (d, *J* = 8.2 Hz, 2H, 17-CH, 19-CH), 5.56 (s, 2H, 9′-NH_2_), 3.67–3.57 (m, 1H, 3′a-CH), 3.53–3.45 (m, 1H, 3′b-CH), 3.22 (t, *J* = 11.5 Hz, 1H, 6-CH), 2.93–2.75 (m, 2H, 5-CH, 7-CH), 2.54 (d, *J* = 11.5 Hz, 1H, 13a-CH), 2.45 (d, *J* = 11.5 Hz, 1H, 13b-CH), 1.96 (m, 2H, 2′-CH_2_), 1.89–1.62 (m, 8H, 2-CH_2_, 4-CH_2_, 10-CH_2_, 11-CH, 1′a-CH), 1.49–1.26 (m, 7H, 3-CH_2_, 8-CH_2_, 9-CH_2_, 1′b-CH); ^13^C-NMR (126 MHz, CDCl_3_) δ 168.2 (7′-C), 161.2 (4′-C), 143.9 (15-C), 133.9 (18-C), 128.1 (17-CH, 19-CH), 125.9 (16-CH, 20-CH), 123.4 (Ph-CF_3_), 62.7 (6-CH), 57.5 (11-CH), 56.6 (2-CH_2_), 56.5 (10-CH_2_), 46.9 (13-CH_2_), 39.7 (7-CH), 34.4 (5-CH), 31.6 (1′-CH_2_), 30.3 (3′-CH_2_), 28.3 (4-CH_2_), 27.9 (8-CH_2_), 25.8 (2′-CH_2_), 20.9 (3-CH_2_), 20.6 (9-CH_2_); ESI-HRMS: *m*/*z* Calcd for C_23_H_31_O_2_N_5_F_3_S_2_ [M + H]^+^, 530.1865; found, 530.1864; HPLC: *t_R_* = 11.41 min, normalization method purity 98.85%.

*12-N-p-Trifluoromethlybenzenesulfonyl-3′-(N-methyl-5-amino-1,3,4-thiadiazol-2-yl)-matrinic propane* (**6g**). Compound **5a** (1.19 g, 2.5 mmol) was treated with 4-methyl-3-thiosemicarbazide (0.26 g, 2.5 mmol) according to the general procedure, then purified by flash column chromatography with dichloromethane/methanol to give the desired product **6g** as a white solid, yield: 45.0%; m.p.: 181 °C (dec.); ${\left[\alpha \right]}_{589}^{25}$ = −1.89; ^1^H-NMR (500 MHz, CDCl_3_) δ 7.97 (d, *J* = 8.2 Hz, 2H, 16-CH, 20-CH), 7.73 (d, *J* = 8.2 Hz, 2H, 17-CH, 19-CH), 5.79 (s, 1H, 9′-NH), 3.68–3.57 (m, 1H, 3′a-CH ), 3.53–3.46 (m, 1H, 3′b-CH), 3.24 (t, *J* = 11.4 Hz, 1H, 6-CH), 3.01 (s, 3H, 9′-NCH_3_), 2.91–2.77 (m, 2H, 5-CH, 7-CH), 2.54 (d, *J* = 11.4 Hz, 1H, 13a-CH), 2.46 (d, *J* = 11.4 Hz, 1H, 13b-CH), 2.02–1.91 (m, 2H, 2′-CH_2_), 1.86–1.63 (m, 8H, 2-CH_2_, 4-CH_2_, 10-CH_2_, 11-CH, 1′a-CH), 1.51–1.31 (m, 7H, 3-CH_2_, 8-CH_2_, 9-CH_2_, 1′b-CH); ^13^C-NMR (126 MHz, CDCl_3_) δ 171.4 (7′-C), 159.2 (4′-C), 144.1 (15-C), 133.9 (18-C), 128.1 (17-CH, 19-CH), 125.8 (16-CH, 20-CH), 123.4 (Ph-CF_3_), 62.7 (6-CH), 57.6 (11-CH), 56.6 (2-CH_2_), 56.5 (10-CH_2_), 47.0 (13-CH_2_), 39.8 (7-CH), 34.5 (5-CH), 33.3 (9′-NCH_3_), 31.6 (1′-CH_2_), 30.4 (3′-CH_2_), 28.3 (4-CH_2_), 28.0 (8-CH_2_), 25.9 (2′-CH_2_), 20.9 (3-CH_2_), 20.7 (9-CH_2_); ESI-HRMS: *m*/*z* Calcd for C_24_H_33_O_2_N_5_F_3_S_2_ [M + H]^+^, 544.2022; found, 544.2019; HPLC: *t_R_* = 11.89 min, normalization method purity 98.90%.

*12-N-p-Trifluoromethlybenzenesulfonyl-3′-(N,N-dimethyl-5-amino-1,3,4-thiadiazol-2-yl)-matrinic propane* (**6h**). Compound **5a** (1.19 g, 2.5 mmol) was treated with 4,4-dimethyl-3- thiosemicarbazide (0.31 g, 2.5 mmol) according to the general procedure, then purified by flash column chromatography with petroleum ether/ethyl acetate to give the desired product **6h** as a white solid, yield: 33.3%; m.p.: 147–148 °C; ${\left[\alpha \right]}_{589}^{25}$ = −3.30; ^1^H-NMR (500 MHz, CDCl_3_) δ 7.97 (d, *J* = 8.2 Hz, 2H, 16-CH, 20-CH), 7.72 (d, *J* = 8.2 Hz, 2H, 17-CH, 19-CH), 3.66–3.62 (m, 1H, 3′a-CH), 3.51 (dd, *J* = 12.6, 6.2 Hz, 1H, 3′b-CH), 3.27–3.22 (m, 1H, 6-CH), 3.09 (s, 6H, 9′-N(CH_3_)_2_), 2.90–2.71 (m, 2H, 5-CH, 7-CH), 2.56–2.53 (m, 1H, 13a-CH), 2.49–2.45 (m, 1H, 13b-CH), 1.99–1.94 (m, 2H, 2′-CH_2_), 1.86–1.61 (m, 8H, 2-CH_2_, 4-CH_2_, 10-CH_2_, 11-CH, 1′a-CH), 1.52–1.29 (m, 7H, 3-CH_2_, 8-CH_2_, 9-CH_2_, 1′b-CH); ^13^C-NMR (126 MHz, CDCl_3_) δ 172.0 (7′-C), 159.3 (4′-C), 144.2 (15-C), 133.8 (18-C), 128.1 (17-CH, 19-CH), 125.8 (16-CH, 20-CH), 123.4 (Ph-CF_3_), 62.8 (6-CH), 57.6 (11-CH), 56.6 (2-CH_2_), 56.5 (10-CH_2_), 47.1 (13-CH_2_), 41.5 (9′N(CH_3_)_2_), 39.8 (7-CH), 34.5 (5-CH), 31.5 (1′-CH_2_), 30.4 (3′-CH_2_), 28.3 (4-CH_2_), 28.0 (8-CH_2_), 25.9 (2′-CH_2_), 20.9 (3-CH_2_), 20.7 (9-CH_2_); ESI-HRMS: *m*/*z* Calcd for C_25_H_35_O_2_N_5_F_3_S_2_ [M + H]^+^, 558.2178; found, 558.2169; HPLC: *t_R_* = 12.71 min, normalization method purity 98.60%.

*12-N-Benzyl-3′-(5-amino-1,3,4-thiadiazol-2-yl)-matrinic propane* (**6i**) Compound **5b** (1.19 g, 2.5 mmol) was treated with thiosemicarbazide (0.23 g, 2.5 mmol) according to the general procedure, then purified by flash column chromatography with dichloromethane/methanol to give the desired product **6i** as a white solid, yield: 34.7%; m.p.: 170–171 °C; ${\left[\alpha \right]}_{589}^{25}$ = −29.79; ^1^H-NMR (600 MHz, CDCl_3_) δ 7.30 (dt, *J* = 15.0, 7.2 Hz, 4H, 16-CH, 17-CH, 19-CH, 20-CH), 7.20 (t, *J* = 7.2 Hz, 1H, 18-CH), 5.67 (s, 2H, 9′-NH_2_), 4.00 (d, *J* = 13.4 Hz, 1H, 14a-CH), 3.12 (d, *J* = 13.4 Hz, 1H, 14b-CH), 2.94–2.72 (m, 5H, 3′-CH_2_, 5-CH, 7-CH, 11-CH ), 2.64 (t, *J* = 11.9 Hz, 1H, 6-CH), 2.33 (m, 1H, 1′a-CH), 1.95–1.57 (m, 12H, 2-CH_2_, 4-CH_2_, 8-CH_2_, 10-CH_2_, 13-CH_2_, 2′-CH_2_), 1.45–1.28 (m, 5H, 3-CH_2_, 9-CH_2_, 1′b-CH); ^13^C-NMR (151 MHz, CDCl_3_) δ 168.3 (7′-C), 161.6 (4′-C), 140.6 (15-C), 128.7 (17-CH, 19-CH), 128.3 (16-CH, 20-CH), 126.7 (18-CH), 64.6 (6-CH), 57.6 (11-CH), 57.4 (14-CH_2_), 57.3 (13-CH_2_), 56.7 (2-CH_2_), 52.4 (10-CH_2_), 37.9 (7-CH), 34.0 (5-CH), 30.8 (1′-CH_2_), 28.3 (3′-CH_2_), 28.2 (4-CH_2_), 27.4 (8-CH_2_), 23.9 (2′-CH_2_), 21.7 (3-CH_2_), 21.4 (9-CH_2_); ESI-HRMS: *m*/*z* Calcd for C_23_H_34_N_5_S [M + H]^+^, 412.2529; found, 412.2530; HPLC: *t_R_* = 8.26 min, normalization method purity 98.65%.

*12-N-Benzyl-3′-(N-methyl-5-amino-1,3,4-thiadiazol-2-yl)-matrinic propane* (**6j**) Compound **5b** (1.19 g, 2.5 mmol) was treated with 4-methyl-3-thiosemicarbazide (0.26 g, 2.5 mmol) according to the general procedure, then purified by flash column chromatography with dichloromethane/methanol to give the desired product **6j** as a white solid, yield: 32.3%; m.p.: 137–138 °C; ${\left[\alpha \right]}_{589}^{25}$ = −28.80; ^1^H-NMR (600 MHz, CDCl_3_) δ 7.32–7.27 (m, 4H, 16-CH, 17-CH, 19-CH, 20-CH), 7.20 (t, *J* = 7.2 Hz, 1H, 18-CH), 5.86 (m, 1H, 9′-NH), 4.01 (d, *J* = 13.0 Hz, 1H, 14a-CH), 3.12 (d, *J* = 13.0 Hz, 1H, 14b-CH), 2.97–2.60 (m, 9H, 3′-CH_2_, 5-CH, 6-CH, 7-CH, 9′-NCH_3_, 11-CH), 2.34 (d, *J* = 11.6 Hz, 1H, 1′a-CH), 1.99–1.52 (m, 12H, 2-CH_2_, 4-CH_2_, 8-CH_2_, 10-CH_2_, 13-CH_2_, 2′-CH_2_), 1.47–1.29 (m, 5H, 3-CH_2_, 9-CH_2_, 1′b-CH); ^13^C-NMR (151 MHz, CDCl_3_) δ 171.4 (7′-C), 159.7 (4′-C), 140.6 (15-C), 128.7 (17-CH, 19-CH), 128.3 (16-CH, 20-CH), 126.7 (18-CH), 64.6 (6-CH), 57.7 (11-CH), 57.4 (14-CH_2_), 57.3 (13-CH_2_), 56.7 (2-CH_2_), 52.3 (10-CH_2_), 37.9 (7-CH), 34.0 (5-CH), 33.3 (9′-NCH_3_), 30.8 (1′-CH_2_), 28.3 (3′-CH_2_), 28.2 (4-CH_2_), 27.4 (8-CH_2_), 24.0 (2′-CH_2_), 21.7 (3-CH_2_), 21.4 (9-CH_2_); ESI-HRMS: *m*/*z* Calcd for C_24_H_36_N_5_S [M + H]^+^, 426.2686; found, 426.2685; HPLC: *t_R_* = 9.14 min, normalization method purity 98.91%.

*12-N-Benzyl-3′-(N,N-dimethyl-5-amino-1,3,4-thiadiazol-2-yl)-matrinic propane hydrochloride* (**6k**). Compound **5b** (1.19 g, 2.5 mmol) was treated with 4,4-dimethyl-3-thiosemicarbazide (0.31 g, 2.5 mmol) according to the general procedure, then purified by flash column chromatography with petroleum ether/ethyl acetate to give the crude product and treated with 2 N hydrochloride/ether (3 mL) to give the desired product **6k** as a white solid, yield: 16.3%; m.p.: 189–190 °C; ${\left[\alpha \right]}_{589}^{25}$ = −14.64; ^1^H-NMR (500 MHz, DMSO-*d*_6_) δ 11.81 (s, 1H, 1-NH^+^), 7.66–7.62 (m, 2H, 16-CH, 20-CH), 7.47–7.43 (m, 3H, 17-CH, 18-CH, 19-CH), 5.00 (d, *J* = 12.7 Hz, 2H, 14-CH_2_), 4.26 (t, *J* = 10.3 Hz, 1H, 6-CH), 3.99–3.93 (m, 2H, 13-CH_2_), 3.61 (d, *J* = 10.3 Hz, 1H, 7-CH), 3.32–3.20 (m, 2H, 3′-CH_2_), 3.05–2.87 (m, 6H, 9′-N(CH_3_)_2_), 2.66–2.60 (m, 2H, 5-CH, 11-CH), 2.57–2.52 (m, 1H, 1′a-CH), 2.18–1.92 (m, 6H, 2-CH_2_, 10-CH_2_, 2′-CH_2_), 1.89–1.55 (m, 8H, 3-CH_2_, 4-CH_2_, 8-CH_2_, 9-CH_2_), 1.43 (d, *J* = 13.6 Hz, 1H, 1′b-CH); ^13^C-NMR (126 MHz, DMSO-*d*_6_) δ 171.0 (7′-C), 158.7 (4′-C), 132.0 (18-C), 130.7 (15-C), 123.0 (17-CH, 19-CH), 129.4 (16-CH, 20-CH), 61.0 (6-CH), 60.8 (14-CH_2_), 58.1 (11-CH), 54.8 (2-CH_2_), 54.7 (10-CH_2_), 49.3 (13-CH_2_), 43.0 (9′-N(CH_3_)_2_), 36.5 (7-CH), 30.5 (5-CH), 29.9 (1′-CH_2_), 28.2 (3′-CH_2_), 26.4 (4-CH_2_), 24.7 (8-CH_2_), 24.2 (2′-CH_2_), 18.5 (3-CH_2_), 18.4 (9-CH_2_); ESI-HRMS: *m*/*z* Calcd for C_25_H_38_N_5_S [M-HCl+H]^+^, 440.2842; found, 440.2839; HPLC: *t_R_* = 9.40 min, normalization method purity 98.49%.

*12-N-p-Methylbenzyl-3′-(5-amino-1,3,4-thiadiazol-2-yl)-matrinic propane* (**6l**) Compound **5c** (0.93 g, 2.5 mmol) was treated with thiosemicarbazide (0.23 g, 2.5 mmol) according to the general procedure, then purified by flash column chromatography with dichloromethane/methanol to give the desired product **6l** as a white solid, yield: 41.4%; m.p.:167–168 °C; ${\left[\alpha \right]}_{589}^{25}$ = −30.62; ^1^H-NMR (600 MHz, CDCl_3_) δ 7.19 (d, *J* = 7.7 Hz, 2H, 16-CH, 20-CH), 7.09 (d, *J* = 7.7 Hz, 2H, 17-CH, 19-CH), 5.68 (s, 2H, 9′-NH_2_), 3.96 (d, *J* = 13.4 Hz, 1H, 14a-CH), 3.08 (d, *J* = 13.4 Hz, 1H, 14b-CH), 2.89–2.73 (m, 4H, 3′-CH_2_, 5-CH, 7-CH), 2.62 (t, *J* = 11.9 Hz, 1H, 6-CH), 2.35–2.32 (m, 1H, 11-CH), 2.31 (s, 3H, Ph-CH_3_), 2.04–2.00 (m, 1H, 1′a-CH), 1.92–1.60 (m, 12H, 2-CH_2_, 4-CH_2_, 8-CH_2_, 10-CH_2_, 13-CH_2_, 2′-CH_2_ ), 1.43–1.29 (m, 5H, 3-CH_2_, 9-CH_2_, 1′b-CH ); ^13^C-NMR (151 MHz, CDCl_3_) δ 168.3 (7′-C), 161.6 (4′-C), 137.3 (18-C), 136.2 (15-C), 129.0 (17-CH, 19-CH), 128.7 (16-CH, 20-CH), 64.6 (6-CH), 57.6 (14-CH_2_), 57.4 (11-CH), 56.4 (2-CH_2_), 52.2 (10-CH_2_), 37.9 (13-CH_2_), 33.9 (7-CH), 30.8 (5-CH), 28.3 (1′-CH_2_), 28.2 (3′-CH_2_), 27.4 (4-CH_2_), 23.9 (8-CH_2_), 21.7 (2′-CH_2_), 21.4 (3-CH_2_), 21.2 (9-CH_2_), 21.1 (Ph-CH_3_); ESI-HRMS: *m*/*z* Calcd for C_24_H_36_N_5_S [M + H]^+^, 426.2686; found; 426.2685; HPLC: *t_R_* =8.78 min, normalization method purity 97.53%.

*12-N-p-Methylbenzyl-3′-(N-methyl-5-amino-1,3,4-thiadiazol-2-yl)-matrinic propane* (**6m**). Compound **5c** (0.93 g, 2.5 mmol) was treated with 4-methyl-3-thiosemicarbazide (0.26 g, 2.5 mmol) according to the general procedure, then purified by flash column chromatography with dichloromethane/methanol to give the desired product **6m** as a white solid, yield: 34.1%; m.p.: 163–164 °C; ${\left[\alpha \right]}_{589}^{25}$ = −35.69; ^1^H-NMR (600 MHz, CDCl_3_) δ 7.19 (d, *J* = 7.6 Hz, 2H, 16-CH, 20-CH), 7.08 (d, *J* = 7.6 Hz, 2H, 17-CH, 19-CH), 6.28 (s, 1H, 9′-NH), 3.96 (d, *J* = 13.3 Hz, 1H, 14a-CH), 3.06 (d, *J* = 13.3 Hz, 1H, 14b-CH), 2.94 (s, 3H, 9′-NCH_3_), 2.89–2.77 (m, 4H, 3′-CH_2_, 5-CH, 7-CH), 2.61 (t, *J* = 12.0 Hz, 1H, 6-CH), 2.35–2.32 (m, 1H, 11-CH), 2.30 (s, 3H, Ph-CH_3_), 2.01 (m, 1H, 1′a-CH), 1.95–1.55 (m, 12H, 2-CH_2_, 4-CH_2_, 8-CH_2_, 10-CH_2_, 13-CH_2_, 2′-CH_2_), 1.44–1.29 (m, 5H, 3-CH_2_, 9-CH_2_, 1′b-CH); ^13^C-NMR (151 MHz, CDCl_3_) δ 171.6 (7′-C), 159.5 (4′-C), 137.4 (18-C), 136.1 (15-C), 128.9 (17-CH, 19-CH), 128.6 (16-CH, 20-CH), 64.6 (6-CH), 57.7 (14-CH_2_), 57.4 (11-CH), 56.4 (2-CH_2_), 52.2 (10-CH_2_), 38.0 (13-CH_2_), 34.0 (7-CH), 33.3 (9′-NCH_3_), 30.8 (5-CH), 28.3 (1′-CH_2_), 28.2 (3′-CH_2_), 27.4 (4-CH_2_), 24.0 (8-CH_2_), 21.7 (2′-CH_2_), 21.4 (3-CH_2_), 21.2 (9-CH_2_), 21.1 (Ph-CH_3_); ESI-HRMS: *m*/*z* Calcd for C_25_H_38_N_5_S [M + H]^+^, 440.2842; found, 440.2845; HPLC: *t_R_* =10.09 min, normalization method purity 95.47%.

*12-N-p-Methylbenzyl-3′-(N,N-dimethyl-5-amino-1,3,4-thiadiazol-2-yl)-matrinic propane hydrochloride* (**6n**). Compound **5c** (0.93 g, 2.5 mmol) was treated with 4,4-dimethyl-3-thiosemicarbazide (0.31 g, 2.5 mmol) according to the general procedure, then purified by flash column chromatography with petroleum ether/ethyl acetate to give the crude product and treated with 2 N hydrochloride/ether (3 mL) to give the desired product **6n** as a white solid, yield: 21.1%; m.p.: 199–200 °C; ${\left[\alpha \right]}_{589}^{25}$ = −17.19; ^1^H-NMR (500 MHz, DMSO-*d*_6_) δ 7.51 (d, *J* = 7.6 Hz, 2H, 16-CH, 20-CH), 7.25 (d, *J* = 7.6 Hz, 2H, 17-CH, 19-CH), 5.76 (s, 1H, 12-NH), 4.94 (m, 1H, 5-CH), 4.24 (m, 1H, 6-CH), 3.98–3.84 (m, 2H, 14-CH_2_), 3.62 (m, 1H, 11-CH), 3.24 (m, 2H, 13-CH_2_), 3.17 (s, 6H, 9′-N(CH_3_)_2_), 3.05–2.86 (m, 4H, 2-CH_2_,10-CH_2_ ), 2.62 (d, *J* = 10.3 Hz, 2H, 3′-CH_2_), 2.32 (s, 3H, Ph-CH_3_), 2.17–2.07 (m, 1H, 7-CH), 1.98 (m, 3H, 1′a-CH, 2′-CH_2_), 1.87–1.55 (m, 8H, 3-CH_2_, 4-CH_2_, 8-CH_2_, 9-CH_2_), 1.43 (d, *J* = 13.8 Hz, 1H, 1′b-CH).; ^13^C-NMR (126 MHz, DMSO-*d*_6_) δ 170.5 (7′-C), 158.0 (4′-C), 138.8 (18-C), 131.4 (15-C), 129.4 (17-CH, 19-CH), 126.9 (16-CH, 20-CH), 60.3 (6-CH), 60.2 (14-CH_2_), 57.2 (11-CH), 55.0 (2-CH_2_), 54.2 (10-CH_2_), 48.5 (13-CH_2_), 42.3 (N(CH_3_)_2_), 35.9 (7-CH), 29.9 (5-CH), 29.2 (1′-CH_2_), 27.6 (3′-CH_2_), 25.8 (4-CH_2_), 24.1 (8-CH_2_), 23.5 (2′-CH_2_), 20.9 (3-CH_2_), 17.9 (9-CH_2_), 17.8 (Ph-CH_3_); ESI-HRMS: *m*/*z* Calcd for C_26_H_40_N_5_S [M − HCl + H]^+^, 454.2999 found, 454.2996; HPLC: *t_R_* = 9.84 min, normalization method purity 96.00%.

*12-N-p-Bromobenzyl-3′-(5-amino-1,3,4-thiadiazol-2-yl)-matrinic propane* (**6o**) Compound **5d** (1.09 g, 2.5 mmol) was treated with thiosemicarbazide (0.23 g, 2.5 mmol) according to the general procedure, then purified by flash column chromatography with dichloromethane/methanol to give the desired product **6o** as a white solid, yield: 44.1%; m.p.: 193–194 °C; ${\left[\alpha \right]}_{589}^{25}$ = −19.58; ^1^H-NMR (500 MHz, CDCl_3_) δ 7.39 (d, *J* = 8.0 Hz, 2H, 16-CH, 20-CH), 7.19 (d, *J* = 8.0 Hz, 2H, 17-CH, 19-CH), 5.61 (s, 2H, 9′-NH_2_), 3.92 (d, *J* = 13.8 Hz, 1H, 14a-CH), 3.06 (d, *J* = 13.8 Hz, 1H,14b-CH), 2.91–2.75 (m, 5H, 3′-CH_2_, 5-CH, 7-CH, 11-CH), 2.64 (t, *J* = 11.8 Hz, 1H, 6-CH), 2.27 (m, 1H, 1′a-CH), 1.94–1.55 (m, 12H, 2-CH_2_, 4-CH_2_, 8-CH_2_, 10-CH_2_, 13-CH_2_, 2′-CH_2_), 1.45–1.30 (m, 5H, 3-CH_2_, 9-CH_2_, 1′b-CH); ^13^C-NMR (126 MHz, CDCl_3_) δ 168.2 (7′-C), 161.5 (4′-C), 139.6 (15-C), 131.4 (17-CH, 19-CH), 130.3 (16-CH, 20-CH), 120.3 (18-C), 64.5 (6-CH), 57.6 (11-CH), 57.4 (14-CH_2_), 57.2 (13-CH_2_), 55.9 (2-CH_2_), 52.3(10-CH_2_), 37.9 (7-CH), 33.9 (5-CH), 30.7(1′-CH_2_), 28.2 (3′-CH_2_), 28.1 (4-CH_2_), 27.4 (8-CH_2_), 23.8 (2′-CH_2_), 21.6 (3-CH_2_), 21.4 (9-CH_2_); ESI-HRMS: *m*/*z* Calcd for C_23_H_33_N_5_BrS [M + H]^+^, 490.1635; found, 490.1635; HPLC: *t_R_* = 9.88 min; normalization method purity 97.24%.

#### 3.2.3. General Procedure for the Synthesis of Compounds **7a–d**


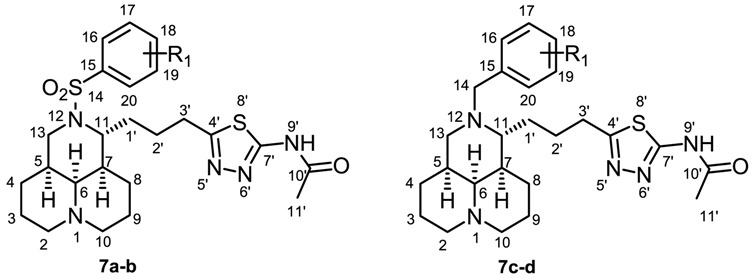


To a solution of **6a**, **6f**, **6i** or **6l** (0.40 mmol) in dichloromethane (10 mL), triethylamine (0.25 g, 2.5 mmol) and acetyl chloride (0.16 g, 2 mmol) were added at 0 °C and stirred at room temperature, until the TLC analysis showed completion of the reaction. The reaction mixture was washed by saturated aqueous ammonium chloride (10 mL × 2) and saturated aqueous sodium chloride (10 mL), dried over anhydrous sodium sulfate and concentrated under reduced pressure. The residue was purified by flash column chromatography on silica gel with dichloromethane/methanol as the eluents to give **7a**–**d**.

*12-N-p-Methylblenzenesulfonyl-3′-(5-acetylamino-1,3,4-thiadiazol-2-yl)-matrinic propane* (**7a**). White solid, yield: 93.7%; m.p.: 273 °C (dec,); ${\left[\alpha \right]}_{589}^{25}$ = −4.83; ^1^H-NMR (500 MHz, DMSO-*d*_6_) δ 12.39 (s, 1H, 9′-NH), 7.68 (d, *J* = 8.0 Hz, 2H, 16-CH, 20-CH), 7.37 (d, *J* = 8.0 Hz, 2H, 17-CH, 19-CH), 3.46–3.38 (m, 2H, 3′-CH_2_), 3.14 (t, *J* = 11.7 Hz, 1H, 6-CH), 2.92–2.84 (m, 1H, 5-CH), 2.83–2.74 (m, 1H, 7-CH), 2.62–2.51 (m, 1H, 11-CH), 2.37 (s, 3H, Ph-CH_3_), 2.17 (s, 3H, 11′CH_3_), 1.96–1.92 (m, 1H, 1a’-CH), 1.89–1.81 (m, 2H, 2′-CH_2_), 1.77–1.66 (m, 8H, 2-CH_2_, 4-CH_2_, 10-CH_2_, 13-CH_2_), 1.58–1.22 (m, 7H, 3-CH_2_, 8-CH_2_, 9-CH_2_, 1′b-CH); ^13^C-NMR (126 MHz, DMSO-*d*_6_) δ 168.4 (7′-C), 163.7 (10′-CO), 158.1 (4′-C), 142.9 (18-C), 136.8 (15-C), 129.5 (17-CH, 19-CH), 127.2 (16-CH, 20-CH), 62.4 (6-CH), 56.7 (11-CH), 56.2 (2-CH_2_), 56.1 (10-CH_2_), 47.2 (13-CH_2_), 38.7 (7-CH), 34.2 (5-CH), 29.6 (1′-CH_2_), 29.0 (3′-CH_2_), 27.5 (4-CH_2_), 27.4 (8-CH_2_), 25.1(2′-CH_2_), 22.4 (11′-CH_3_), 21.0 (Ph-CH_3_), 20.4 (3-CH_2_), 20.3 (9-CH_2_); ESI-HRMS: *m*/*z* Calcd for C_25_H_36_O_3_N_5_S_2_ [M + H]^+^, 518.2254; found, 518.2252; HPLC: t*_R_* = 11.66 min, normalization method purity 95.35%.

*12-N-p-Trifluoromethlybenzenesulfonyl-3′-(5-acetylamino-1,3,4-thiadiazol-2-yl)-matrinic propane* (**7b**). White solid, yield: 99.1%; m.p.: 197 °C (dec,); ${\left[\alpha \right]}_{589}^{25}$ = −1.30; ^1^H-NMR (600 MHz, CDCl_3_) δ 13.27 (s, 1H, 9′-NH), 7.98 (d, *J* = 8.2 Hz, 2H, 16-CH, 20-CH), 7.73 (d, *J* = 8.2 Hz, 2H, 17-CH, 19-CH), 3.66–3.57 (m, 1H, 3′a-CH), 3.53–3.42 (m, 1H, 3′b-CH), 3.25 (t, *J* = 11.6 Hz, 1H, 6-CH), 3.03–2.91 (m, 2H, 5-CH, 7-CH), 2.54 (d, *J* = 11.4 Hz, 1H, 13a-CH), 2.49–2.42 (m, 4H, 13b-CH, 11′-CH_3_), 1.99–1.96 (m, 2H, 2′-CH_2_), 1.88–1.74 (m, 8H, 2-CH_2_, 4-CH_2_, 10-CH_2_, 11-CH, 1′a-CH), 1.52–1.29 (m, 7H, 3-CH_2_, 8-CH_2_, 9-CH_2_, 1′b-CH); ^13^C-NMR (151 MHz, CDCl_3_) δ 168.9 (7′-C), 164.6 (10′-CO), 160.5 (4′-C), 144.1 (15-C), 134.0 (18-C), 128.1 (17-CH, 19-CH), 125.8 (16-CH, 20-CH), 123.4 (Ph-CF_3_), 62.7 (6-CH), 57.5 (11-CH), 56.7 (2-CH_2_), 56.6 (10-CH_2_), 46.9 (13-CH_2_), 39.8 (7-CH), 34.4 (5-CH), 31.8 (1′-CH_2_), 29.8 (3′-CH_2_), 28.4 (4-CH_2_), 28.0 (8-CH_2_), 25.8 (2′-CH_2_), 23.3 (11′-CH_3_), 20.9 (3-CH_2_), 20.7 (9-CH_2_); ESI-HRMS: *m*/*z* Calcd for C_25_H_33_O_3_N_5_F_3_S_2_ [M + H]^+^, 572.1971; found, 572.1970; HPLC: t*_R_* =11.91 min, normalization method purity 99.53%.

*12-N-Benzyl-3′-(5-acetylamino-1,3,4-thiadiazol-2-yl)-matrinic propane* (**7c**). White solid, yield: 93.5%; m.p.: 188–189 °C; ${\left[\alpha \right]}_{589}^{25}$ = −27.65; ^1^H-NMR (500 MHz, CDCl_3_) δ 13.31 (s, 1H, 9′-NH), δ 7.32–7.27 (m, 4H, 16-CH, 17-CH, 19-CH, 20-CH), 7.20 (t, *J* = 7.2 Hz, 1H, 18-CH), 3.98 (d, *J* = 13.4 Hz, 1H, 14a-CH), 3.10 (d, *J* = 13.4 Hz, 1H, 14b-CH), 2.97–2.73 (m, 5H, 3′-CH_2_, 5-CH, 7-CH, 11-CH), 2.63 (t, *J* = 12.0 Hz, 1H, 6-CH), 2.42 (s, 3H, 11′-CH_3_), 2.37–2.31 (m, 4H, 2-CH_2_,10-CH_2_), 2.06–1.78 (m, 8H, 4-CH_2_, 8-CH_2_, 13-CH_2_, 2′-CH_2_), 1.47–1.22 (m, 6H, 3-CH_2_, 9-CH_2_, 1′-CH_2_); ^13^C-NMR (126 MHz, CDCl_3_) δ 169.0 (7′-C), 165.0 (10′-CO), 160.5 (4′-C), 140.4 (15-C), 128.6 (17-CH, 19-CH), 128.4 (16-CH, 20-CH), 126.8 (18-CH), 64.6 (6-CH), 57.6 (11-CH), 57.4 (14-CH_2_), 56.9 (13-CH_2_), 56.7 (2-CH_2_), 52.4 (10-CH_2_), 37.9 (7-CH), 34.0 (5-CH), 30.2 (1′-CH_2_), 28.4 (3′-CH_2_), 28.2 (4-CH_2_), 27.4 (8-CH_2_), 23.9 (11′-CH_3_), 23.3 (2′-CH_2_), 21.6 (3-CH_2_), 21.4 (9-CH_2_); ESI-HRMS: *m*/*z* Calcd for C_25_H_36_ON_5_S [M + H]^+^, 454.2662; found, 454.2662; HPLC: t*_R_* = 8.96 min, normalization method purity 98.36%.

*12-N-p-Methylbenzyl-3′-(5-acetylamino-1,3,4-thiadiazol-2-yl)-matrinic propane* (**7d**). White solid, yield: 91.5%; m.p.: 226 °C (dec.); ${\left[\alpha \right]}_{589}^{25}$ = −31.74; ^1^H-NMR (500 MHz, CDCl_3_) δ 13.34 (s, 1H, 9′-NH), 7.19 (d, *J* = 7.6 Hz, 2H, 16-CH, 20-CH), 7.09 (d, *J* = 7.6 Hz, 2H, 17-CH, 19-CH), 3.98 (d, *J* = 13.3 Hz, 1H, 14a-CH), 3.10 (d, *J* = 13.3 Hz, 1H, 14b-CH), 2.95–2.86 (m, 2H, 3′-CH_2_), 2.83 (m, 1H, 5-CH), 2.76 (m, 1H, 7-CH), 2.63 (t, *J* = 12.0 Hz, 1H, 6-CH), 2.42 (s, 3H, 11′-CH_3_), 2.36 (d, *J* = 11.0 Hz, 1H, 11-CH), 2.31 (s, 3H, Ph-CH_3_), 2.03 (s, 1H, 1′a-CH), 1.92–1.78 (m, 6H, 2-CH_2_, 4-CH_2_, 8-CH_2_,), 1.75–1.62 (m, 4H, 10-CH_2_, 2′-CH_2_ ), 1.48–1.30 (m, 6H,3-CH_2_, 9-CH_2_, 13-CH_2_), 1.25 (s, 1H, 1′b-CH); ^13^C-NMR (126 MHz, CDCl_3_) δ 169.0 (7′-C), 165.1 (10′-CO), 160.5 (4′-C), 137.4 (18-C), 136.2 (15-C), 129.0 (17-CH, 19-CH), 128.6 (16-CH, 20-CH), 64.6 (6-CH), 57.7 (14-CH_2_), 57.5 (11-CH), 56.7 (2-CH_2_), 52.4 (10-CH_2_), 38.0 (13-CH_2_), 34.1 (7-CH), 30.2 (5-CH), 29.8 11′-CH_3_), 28.5 (1′-CH_2_), 28.2 (3′-CH_2_), 27.4 (4-CH_2_), 24.0 (8-CH_2_), 23.3 (2′-CH_2_), 21.7 (3-CH_2_), 21.5 (9-CH_2_), 21.2 (Ph-CH_3_); ESI-HRMS: *m*/*z* Calcd for C_26_H_38_ON_5_S [M + H]^+^, 468.2792; found, 468.2789; HPLC: t_R_ =9.34 min, normalization method purity 98.77%.

### 3.3. Biology Assay

#### 3.3.1. Cell Culture and Screening of Compound

Cells were laid on 96-well plate, cultured in Dulbecco’s Modified Eagle’s medium (DMEM), containing 10% fetal bovine serum (FBS) in a 5% CO_2_ atmosphere at 37 °C. Moreover, serum-free culture was required until the cells at 90–95% confluence. After 24 h, cells were treated with a matrinic derivative (80 μM) for 24 h. The COL1A1 promotor activity was determined using the Bright-Glo luciferase assay system (Promega Corporation, Madison, WI, USA) [8].

#### 3.3.2. Cell Survival Assay

The cell survival was evaluated by MTT assay. HepG2 cells were seeded at density of 6 × 10^3^ cells/well in 96-well plate. Cells were treated with various concentrations of matrinic derivate until the cells at 50% confluence. After 24 h of incubation, 20 µL of the MTT (5 mg/mL) solution was added into each plate and incubated for 4 h at 37 °C, 5% CO_2_. Subsequently, the culture supernatant was replaced with 150 µL DMSO to dissolve the formazan crystal. The absorbance at 570 nm was measured using a microplate reader (ELx800, BioTek Instruments, Winooski, VT, USA).

#### 3.3.3. RT-qPCR Assay

LX-2 cells were seeded in 6-well plate, cultured in DMEM, containing 10% fetal bovine serum (FBS) in a 5% CO_2_ atmosphere at 37 °C. And serum-free culture was required until the cells at 90–95% confluence. After 24 h, cells were treated with TGFβ1 (2 ng/mL) and matrinic derivatives (80 μM) for 24 h. Total RNA from the LX-2 cells was extracted using Trizol reagent, purified by NucleoSpin RNA Clean-up. Reverse transcription was performed with a Transcriptor first strand cDNA synthesis kit. The cDNA was then analysis by ABI 7500 Fast Real-Time PCR System (ThermoFisher Scientific, Singapore) using TaqMan probes of TGFβ1, COL1A1, α-SMA, and GAPDH (sequence reserved by ABI, Foster City, CA ,USA) and FastStart Universal Probe master mix (Roche, Indianapolis, IN, USA).

#### 3.3.4. Western Blot

LX-2 cells were cultured as described above. Briefly, cells were washed with phosphate-buffered saline (PBS) and were lysed in radioimmunoprecipitation assay (RIPA) lysis for 30 min in 4 °C; the supernatant was collected after centrifugation at 12,000× *g*, 4 °C for 15 min. Equal amounts of protein were quantified with Bradford assay, separated by SDS-PAGE and transferred to polyvinylidene difluoride membrane. The membranes were blocked for one hour at room temperature in PBST containing 5% milk and probed with specific first antibodies overnight at room temperature. Membrane was washed 3 times by PBST, followed by horseradish peroxidase-conjugated secondary antibodies and GAPDH. The proteins were visualized using chemiluminescence reagents.

### 3.4. Acute Toxicity

Female Kunming mice with weight of 20.0 (±1.0 g) were obtained from the Institute of Laboratory Animal Science (Beijing, China). Animals were cared according to the institutional guidelines of the Institute of Materia Medica, CAMS&PUMC (00000364). The mice were fed with regular rodent chow and housed in an air conditioned room. The mice were randomly divided into different groups with 6 mice each. Each compound was given orally in a single-dosing experiment at 0, 250, 500, 750 or 1000 mg·kg^−1^ (ddH_2_O as control), respectively. The mice were closely monitored for 7 days. Body weight as well as survival was monitored.

## 4. Conclusions

To summarize, a series of novel matrinic thiadiazole derivatives were designed, synthesized and evaluated for their inhibitory effect on the COL1A1 promotor. The SAR indicated that: (i) the introduction of a thiadiazole on the 11-side chain was beneficial for the activity; (ii) a 12-*N*-benzyl was favorable for activity. Among the new derivatives, compound **6n** gave a high inhibitory effect on COL1A1 promotor at a rate of 39.7% on the cellular level at a concentration of 40 μM. It also effectively inhibited the expression of two representative fibrogenetic collagen proteins (COL1A1 and α-SMA) on both the mRNA and protein levels as well as a high safety profile with the LD_50_ value of over 500 mg·kg^−1^ in vivo. Further study indicated that it might exert anti-liver fibrotic activity via repressing the TGFβ/Smad pathway. Overall, this results offer powerful information for further structure optimization, in which compound **6n** has been chosen as an ideal anti-liver fibrosis lead for further investigation by means of computer aided drug and other design strategies.

## Figures and Tables

**Table 1:**
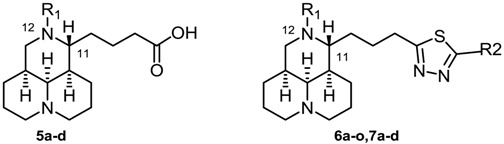
Inhibition of COL1A1 promotor of target compounds and cytotoxicity of key compounds.

Code	R_1_	R_2_	Inhibition Percentage *^a^*	CC_50_ (μM) *^b^*
**1**	*p*-CH_3_C_6_H_4_SO_2_	-	18.5% ± 1.1%	>320
**5a**	*p*-CF_3_C_6_H_4_SO_2_	-	−3.7% ± 3.0%	NT *^c^*
**5b**	C_6_H_5_CH_2_	-	−33.6% ± 10.6%	NT *^c^*
**5c**	*p*-CH_3_C_6_H_4_CH_2_	-	−116.2% ± 45.7%	NT *^c^*
**5d**	*p*-BrC_6_H_4_CH_2_	-	6.4%± 0.9%	NT *^c^*
**6a**	*p*-CH_3_C_6_H_4_SO_2_	NH_2_	21.2% ± 10.4%	>320
**6b**	*p*-CH_3_C_6_H_4_SO_2_	NHCH_3_	12.8% ± 7.3%	NT *^c^*
**6c**	*p*-CH_3_C_6_H_4_SO_2_	NHCH(CH_3_)_2_	−84.2% ± 63.1%	NT *^c^*
**6d**	*p*-CH_3_C_6_H_4_SO_2_	N(CH_3_)_2_	−36.7% ± 17.3%	NT *^c^*
**6e**	*p*-CH_3_C_6_H_4_SO_2_	NHPh	69.0% ± 13.5%	82.55
**6f**	*p*-CF_3_C_6_H_4_SO_2_	NH_2_	−19.0% ± 7.8%	NT *^c^*
**6g**	*p*-CF_3_C_6_H_4_SO_2_	NHCH_3_	6.1% ± 0.8%	NT *^c^*
**6h**	*p*-CF_3_C_6_H_4_SO_2_	N(CH_3_)_2_	−35.2% ± 2.1%	NT *^c^*
**6i**	C_6_H_5_CH_2_	NH_2_	47.8% ± 29.0%	>320
**6j**	C_6_H_5_CH_2_	NHCH_3_	29.5% ± 6.1%	NT *^c^*
**6k**	C_6_H_5_CH_2_	N(CH_3_)_2_	15.2% ± 7.1%	NT *^c^*
**6l**	*p*-CH_3_C_6_H_4_CH_2_	NH_2_	38.7% ± 4.4%	>320
**6m**	*p*-CH_3_C_6_H_4_CH_2_	NHCH_3_	−29.6% ± 13.6%	NT *^c^*
**6n**	*p*-CH_3_C_6_H_4_CH_2_	N(CH_3_)_2_	39.7%± 12.3%	>320
**6o**	*p*-BrC_6_H_4_CH_2_	NH_2_	7.8% ± 3.0%	NT *^c^*
**7a**	*p*-CH_3_C_6_H_4_SO_2_	NHCOCH_3_	33.9% ± 9.9%	NT *^c^*
**7b**	*p*-CF_3_C_6_H_4_SO_2_	NHCOCH_3_	−6.0% ± 5.0%	NT *^c^*
**7c**	C_6_H_5_CH_2_	NHCOCH_3_	12.9% ± 10.6%	NT *^c^*
**7d**	*p*-CH_3_C_6_H_4_CH_2_	NHCOCH_3_	27.0% ± 9.5%	NT *^c^*
EGCG	-	-	27.5% ± 7.9%	-
DMSO	-	-	2.9% ± 0	-

*^a^* The inhibition rate of COL1A1 promotor’s activity in LX2 cells; *^b^* Cytotoxic concentration required to inhibit HepG2 cells cell growth by 50%; *^c^* Not tested.
